# The impact of sodium reduction on overall nutrient content in Child and Adult Care Food Program meals

**DOI:** 10.1017/S1368980023001167

**Published:** 2023-11

**Authors:** Elise Gahan, Elinor Hansotte, K Elise Lindstrom, Shelley Vaughn, Sandra Cummings

**Affiliations:** Marion County Public Health Department, 3838 Rural Street, Indianapolis, IN 46205, USA

**Keywords:** Child and Adult Care Food Program, Na reduction, food service guidelines

## Abstract

**Objective:**

To understand the impact of Na reduction on the nutrient content of Child and Adult Care Food Program (CACFP) meals served through At-Risk Afterschool Meals (ARASM) without compromising the nutritional quality of the meals served.

**Design:**

Sodium Reduction in Communities Program (SRCP) partnered with a CACFP ARASM programme from October 2016 to September 2021. We assessed changes in Healthy Eating Index 2015 (HEI-2015) food component scores and macro- and micronutrients using cross-sectional nutrient analyses of October 2016 and 2020 menus.

**Setting:**

ARASM programme sites in Indianapolis, IN, USA.

**Participants:**

October 2016 and 2020 menus from one CACFP ARASM programme.

**Intervention:**

Na reduction strategies included implementing food service guidelines, modifying meal components, changing procurement practices and facilitating environmental changes to promote lower Na items.

**Results:**

From baseline in 2016 to 2020, fifteen meal components were impacted by the intervention, which impacted 17 (85 %) meals included in the analysis. Average Na per meal reduced significantly between 2016 (837·9 mg) and 2020 (627·9 mg) (*P* = 0·002). Between 2016 and 2020, there were significant increases in whole grains (*P* = 0·003) and total vegetables (*P* < 0·001) and significant reductions in refined grains (*P* = 0·001) and Na (*P* = 0·02), all per 1000 kcal served.

**Conclusions:**

This study demonstrates that Na content can be reduced in CACFP meals without compromising the nutritional quality of meals served. Future studies are needed to identify feasible best practices and policies to reduce Na content in the CACFP meal pattern.

The overall diet quality of school-age children in the USA falls below recommended guidelines^(1)^. In general, low-income children’s diets are less nutritious than their higher-income counterparts^(2,3)^. Consuming a lower-quality diet over time is a risk factor for developing diet-related diseases such as type 2 diabetes, high blood pressure and heart disease^(4–7)^. Because adequate nutrition is essential to healthy growth and development, the United States Department of Agriculture operates several Child Nutrition Programs for school-age children including the School Breakfast Program, the National School Lunch Program, Summer Food Service Program and the Child and Adult Care Food Program (CACFP). Meals are reimbursable if they meet all programme requirements. The CACFP meal pattern was revised in April 2016, and the deadline for compliance was October 1, 2017. The following updates sought to better align meals with the Dietary Guidelines for Americans: separating fruits and vegetables into individual meal components; allowing CACFP providers the flexibility to serve two vegetables at the lunch and supper meal instead of one fruit and one vegetable at both meals; requiring at least one grain serving a day to be whole grain rich and disqualifying grain-based desserts from reimbursement. Cereal must contain no more than 6 grams of sugar per dry ounce, and yogurt must contain no more than 23 g of sugar per six ounces^(8)^. These changes were meant to increase the amount of fruits, vegetables and whole grain-rich foods eaten, which may increase commonly under-consumed nutrients including fibre, potassium, vitamins D and E and reduce overconsumed nutrients such as saturated fats and added sugars^(9)^.

CACFP provides snacks and meals to children and adults at qualifying childcare centers, in-home daycares, afterschool programmes and adult daycare centers. Reimbursed meals must follow a component-based meal pattern (Table 1). Snacks must include at least two components and meals must include all five components. Afterschool programmes where at least half of the children qualify for free and reduced-priced school meals and who offer enrichment activities can serve meals and snacks through the At-Risk Afterschool Meals (ARASM) Program. ARASM fills an important nutrition gap for participants; 1·471 million school-age children received after-school snack and supper meals in 2020^(10)^.

**Table 1:** CACFP meal component requirements for children aged 6–12

Component	Requirement, aged 6–12
Milk	1 cup
Meat and meat alternative	2 ounces
Vegetables	1/2 cup
Fruit	1/2 cup
Grain	1 ounce equivalent

The Healthy Eating Index-2015 (HEI-2015) assesses how well aligned a diet pattern is with the recommendations of the Dietary Guidelines for Americans. The overall score is made up of thirteen food components and can range from 0 to 100. A higher score represents stronger alignment with the Dietary Guidelines for Americans^(11)^. Due to the CACFP requirements, it is not possible for the score of this analysis to be 0. A National Health and Nutrition Examination Survey (NHANES) found that diet quality is higher for young children than it is for school-age children and adolescents. Using the HEI-2015, children aged 2–4 scored 61 points, children aged 5–8 scored 55 points, children aged 9–13 scored 52 and children aged 14–18 scored 51 points^(1)^.

Na, one of the HEI-2015 components of moderation, is overconsumed by most Americans, including children. The average Na intake for children aged 6–18 is 3256 mg^(12)^. The upper limit for children aged 1–3 is 1200 mg per day, 1500 mg per day for children aged 4–8, 1800 mg per day for children aged 9–13 and 2300 mg per day for adolescents aged 14 and older^(1)^. Almost 90 % of children and adolescents exceed the upper limit daily^(12)^. Consuming too much Na can contribute to hypertension and adverse cardiovascular events including heart attacks and stroke^(13)^. The prevalence of hypertension in youth aged 12–19 was 4·2 % using 2013–2016 NHANES data^(14)^. Maintaining daily Na consumption at or below the recommended limits in childhood can reduce CVD risk in adulthood^(15)^. Meals served through CACFP often exceed Na recommendations even after the meal pattern update^(16–19)^.

The Centers for Disease Control and Prevention (CDC) funded the Sodium Reduction in Communities Program (SRCP) to address the overconsumption of Na across the lifespan. The CDC awarded Marion County Public Health Department (MCPHD) (Marion County, Indiana) a 5-year (October 2016 through September 2021) cooperative agreement to implement SRCP. MCPHD partnered with Indianapolis Parks and Recreation’s (Indy Parks) ARASM programme and its food service company to reduce Na. The ARASM programme serves prepackaged, cold meals composed of CACFP meal components that must be individually packaged for food safety and to enable meal service in venues without the capacity to hot hold foods, such as parks and libraries. Na reduction strategies included implementing food service guidelines and nutritional standards for Na content for meals and individual meal components, modifying meal components to reduce Na content, improving procurement practices and facilitating environmental changes to promote lower Na items^(20)^. The MCPHD Registered Dietitian Nutritionists (RDN) and Indy Parks food programme staff worked together to create Na guidelines for meals and individual meal components (Table 2). Meals and meal components with a Na level at or below the guideline were considered ‘lower sodium.’

**Table 2:** Indy parks SRCP Na guidelines by Child and Adult Care Food Program (CACFP) meal component

CACFP meal component	Na guideline (mg)
Meals	740
Meat/Alternative	480
Grain	230
Vegetable	230
Fruit	230

Evaluations of menus have demonstrated that childcare providers have largely been able to successfully implement the revised CACFP meal plan^(19,21–23)^. However, most of the existing research has focussed on younger children in early childhood education centers and examined adherence to the revised meal pattern, not the nutrient composition of meals, including Na. The objective of this study was to examine the impact of implementing Na reduction strategies in a CACFP programme on the overall nutrient profile of meals from baseline in October 2016 to the final intervention year in October 2020.

## Methods

### Population

Indy Parks’ ARASM served 209 467 meals in 2016 and 133 996 meals in 2020. The decline in the number of meals served was due to COVID-19 programme disruptions. In 2016, one meal was served daily Monday–Saturday. In 2020, one meal was served daily Monday–Friday. Meals were served at forty-nine sites in both 2016 and 2020. Snacks were served in this programme but were excluded from the current study’s analysis.

### Intervention

The SRCP cooperative agreement began on October 1, 2016. An Advisory Team was created that included project partners (Indy Parks, their food service company administrator, chef and key personnel) and the MCPHD SRCP staff which included two RDN, an evaluator and a project manager. The food service company was enthusiastic about the intervention and entered into a deliverables-based contract that included payment to offset staff’s time spent on the project and to ensure data was provided. The Advisory Team first met in January 2017 to plan for data collection. The baseline nutrient analysis of the 4-week October 2016 menu cycle was completed in April 2017. The Advisory Team determined high-Na foods and meals to modify and identified acceptable lower Na products for substitution. SRCP interventions began in summer 2017. Quarterly Advisory Team Meetings continued for duration of the cooperative agreement. The Na reduction strategies were implemented reiteratively throughout the cooperative agreement. Menu analyses were conducted annually, meals and meal components with high Na content were targeted for intervention, the RDN and food service company identified alternatives that met nutritional and cost needs and new food items were taste tested to obtain child feedback.

### Data collection

SRCP staff collected ARASM 4-week cycle menus annually for the duration of the 5-year project. The RDN met with the food service company to obtain brand names and the nutrition facts label information for each product. The CACFP meal pattern for children aged 6–12 was used. The baseline menus were from October 2016 before the implementation of both the revised CACFP meal pattern and MCPHD’s SRCP intervention. A 4-week menu cycle consisting of 5 weekdays and 1 weekend meal, for a total of twenty-four meals, was used. Year 5 menus were from October 2020. A 4-week menu cycle consisting of 5 weekday meals, for a total of twenty meals, was used. Reflecting CACFP meal pattern updates, all twenty meals included in the 2020 data set included 1 % milk, a fruit, a vegetable, a meat or meat alternative and a whole grain product. The cycle menus were repeated every 4 weeks throughout the year. There were no seasonal adjustments. Meals and meal components that were impacted by SRCP interventions were tracked on a product replacement log (Table 3). SRCP staff conducted taste tests on select lower Na food replacements to gauge child acceptance.

**Table 3:** Product replacement log

Product	Na content (mg)	Intervention strategy	Date implemented	Na difference (mg)	Na change (%)	Cost difference	Cost change (%)
Bologna and Cheese on Enriched Bun	960	Product Swap	Summer 2017	−270	−28 %	–$0·03	−4 %
Turkey Bologna, Turkey Salami, Cheese on Whole Grain Sub	690						
Turkey and Cheese on Enriched Bun	990	Product Swap	Summer 2017	−280	−28 %	–$0·18	−17 %
Turkey and Cheese on Whole Grain Sub	710						
Ham and Cheese on Enriched Bun	1010	Product Swap	Summer 2017	−570	−56 %	–$0·01	−1 %
Turkey Bologna and Cheese on Whole Grain Bun	440						
Granola Bar	120	Product Swap	Fall 2017	−30	−25 %	–$0·03	−11 %
Corn Bread	90						
Enriched White 4" Bun	240	Product Swap	Winter 2018	−125	−52 %	–$0·02	−15 %
Low Na Whole Grain 3·5" Bun	115						
Individually Wrapped Whole Wheat Bread Slice	184	Product Swap	Fall 2018	−69	−38 %	–$0·07	−29 %
Reduced Na Individually Wrapped Whole Wheat Bread Slice	115						
Cheese Stick – Baseline	200	Product Swap	Spring 2019	−50	−25 %	$0·08	31 %
Cheese Stick – Final	150						
Peanut Butter and Jelly Sandwich	320	Product Swap	Fall 2019	−120	−38 %	–$0·09	−13 %
Whole Grain Wowbutter and Grape Jelly Sandwich	200						
Whole Grain Chicken Tenders (390 mg), Barbeque Sauce (90 mg)	480	Product Swap	Spring 2020	−90	−19 %	–$0·06	−8 %
Barbeque Chicken Breast	390						
Enriched White 10" Tortilla	390	Product Swap	Summer 2020	−240	−62 %	$0·04	20 %
Ultra Grain 10" Tortilla	150						
Pork Chop (384 mg), Mustard (85 mg), Enriched Bun (240 mg)	709	Meal Replacement	Summer 2019	−219	−31 %	$0·26	28 %
Taco Hummus (200 mg), Cheddar Cheese Stick (180 mg) Tortilla Chips (110 mg)	490						
Riblet (740 mg), Enriched Bun (240 mg)	980	Meal Replacement	Spring 2019	−480	−50 %	$0·53	75 %
Taco Hummus (200 mg), Cheddar Cheese Stick (180 mg), Tortilla Chips (110 mg)	490						
Whole Grain Chicken Patty (400 mg), Mustard (85 mg), Enriched Bun (240)	725	Meal Replacement	Spring 2020	−355	−49 %	$0·39	63 %
Cinnamon Chex (170 mg), Yogurt (50 mg), Cheese (150 mg)	370						
Beef Wonder Bites (525 mg), Whole Wheat Bread (184 mg)	709	Meal Replacement	Summer 2020	−339	−48 %	–$0·11	−10 %
Cinnamon Chex (170 mg), Yogurt (50 mg), Cheese (150 mg)	370						
Strawberry Yogurt Chex (55 mg), Yogurt (50 mg), Cheese Stick (180 mg)	285	Meal Addition	Spring 2020				

As a robustness check, we developed a mock 2016 dataset that accounted for changes that would have been required by the 2017 CACFP meal pattern update regardless of SRCP intervention. The mock 2016 dataset maintained the nutrition information for true baseline foods except for those that did not comply with the meal pattern update. The authors assume that these changes would have been made to the meals due to CACFP requirements regardless of SRCP intervention, so the mock 2016 dataset allows for an analysis of true SRCP influence. In 2016, a meat or meat alternative and 1 % milk were served in all twenty-four meals. A fruit was served with twenty-three meals, a vegetable was served with twenty meals and a whole grain was served with twelve meals. Five meals did not serve both a fruit and a vegetable; of those, four meals contained two fruits and one meal contained two vegetables. An enriched bun accounted for the eleven grains that were not considered whole grains. The mock 2016 dataset changed the eleven times the enriched bun was served to a whole grain bun and modified the four meals that served two fruits to serving one fruit and one vegetable, resulting in vegetables served all 24 days. The revised CACFP meal pattern allows two vegetables to be served in a meal, so the 1 day that served two vegetables remained as two vegetables. In accordance with the CACFP update, all twenty-four mock 2016 dataset meals contained 1 % milk, a fruit (or two vegetables), a vegetable, a meat or meat alternative and a whole grain. No additional changes were made in the mock 2016 menu.

### Analysis

This study analysed the HEI-2015 food components and nutrient profile of ARASM menus in 2016 and 2020 after SRCP intervention. To analyse an expanded set of nutrients, the RDN entered products into the nutrition software programme Nutrient Data Systems for Research (NDSR), Version 51, 2020, which was developed by the Nutrition Coordinating Center (NCC) (University of Minnesota, Minneapolis, MN)^(24)^. NDSR uses nutrient information about each product to analyse meals by a host of nutrients as well as food components. The RDN compared the nutrition facts label and NDSR output for completeness and consistency. Adjustments were made to the NDSR input until the best match for each food item was achieved (e.g. if the specific brand of certain foods was not available within the NDSR library, a best match based on nutrient profile was used). If there was not a food product in NDSR that fit the nutrition facts label according to NDSR guidelines (Table 4), a new food request was submitted to NDSR. Meal components were analysed in ounce equivalents per 1000 kcal for grain and protein and in cup equivalents per 1000 kcal for dairy, fruit and vegetables.

**Table 4:** Nutrient Data Systems for Research nutrient variation tolerances per 100 g product

Nutrient	Variation tolerance per 100 g
Calories	+/– 355·64 kJ (85 kcal)
Protein	+/– 5·00 g
Total Fat	+/– 2·50 g
Total carbohydrate	+/– 10·00 g
Na	+/– 100·00 mg

Included in the NDSR software is a set of proprietary standardised SAS codes that are used for the analysis of HEI-2015 food components. Most HEI-2015 food components are analysed by density, using servings per 1000 kcal. Fatty acids are analysed as a ratio of PUFA and unsaturated fatty acids to SFA and added sugars and saturated fats are analysed as a percentage of total energy. HEI-2015 adequacy components included in this study were total fruit, whole fruit, total vegetables, dark green vegetables, whole grains, dairy, total protein foods, seafood and plant protein and fatty acids. HEI-2015 moderation components included in this study were refined grains, Na, added sugars and saturated fats. For this study, macro- and micronutrients were calculated in grams or micrograms. The means of specified nutrition variables and HEI-2015 food components were compared using *t* tests to determine if there was a significant difference between each nutrient or food component from 2016 to 2020. The level of significance was set at *P* < 0·05. As the HEI-2015 component or total scores consider all foods eaten in a day, they were not calculated for this analysis of one daily meal.

SAS Enterprise 7.1 (SAS Institute, Inc.) was used for SRCP and HEI nutrient analyses.

## Results

There were ten product swaps, four meal replacements and one meal addition during the SRCP intervention as documented in the product replacement log (Table 3). In 2016, twelve out of twenty-four meals met the Na guidelines. Seventeen out of twenty meals were impacted by an intervention strategy in 2020. As a result of the intervention strategies, seventeen out of twenty meals met the Na guidelines. Changes were labour neutral for the food service company, and they were able to sustain the cost changes within CACFP reimbursement rates. Pre/post-taste tests were conducted on two product replacements and found that the children rated the replacement higher for both. Acceptance-only surveys were completed for three lower Na food items, and the children rated all products above the pre-set acceptability level.

The average Na per meal decreased significantly between 2016 and 2020 from 837·9 mg to 627·9 mg (*P* < 0·05), a 25 % reduction. The change in kJ per meal was not considered significant but decreased from an average of 2254.34 kJ to 2095.35 kJ (538.8 kcal to 500.8 kcal), a 7 % reduction (See Table 5). In our robustness check (Table 6), the average Na per meal maintained a significant decrease (825·5 mg to 627·9 mg (*P* < 0·05)) between the mock 2016 dataset and 2020 dataset. The change in kJ per meal was not considered significant (2170.24 kJ to 2095.35 kJ, 518.7 kcal to 500.8 kcal).

**Table 5:** Comparison of macro- and micronutrients from 2016 to 2020 menu

Nutrient	Mean serving 2016 menu	sd	Mean serving 2020 menu	sd	*P*-value
Energy (kJ)	2254.34	239.07	2095.35	297.19	0·06
Protein (g)	25·86	2·99	24·71	3·29	0·23
Carbohydrate (g)	68·57	8·17	62·74	8·61	0·03*
Dietary fibre (g)	4·79	2·01	5·67	1·69	0·13
Sugar (g)	37·93	9·64	35·64	9·05	0·42
Added sugar (g)	7·59	4·58	6·61	8·43	0·65
Total fat (g)	19·07	5·51	18·27	7·11	0·68
Saturated fat (g)	6·61	2·01	6·46	1·56	0·79
Monounsaturated fat (g)	5·86	2·17	4·79	1·42	0·07
Polyunsaturated fat (g)	5·04	3·57	5·62	4·17	0·63
Trans fat (g)	0·36	0·20	0·37	0·08	0·86
Cholesterol (mg)	51·80	21·21	41·34	8·81	0·04*
Na (mg)	837·93	237·26	627·88	164·65	0·002*
Vitamin A (mcg)	340·35	247·25	501·28	275·87	0·05*
Vitamin C (mg)	24·53	31·44	23·87	26·73	0·94
Vitamin D (mcg)	3·25	0·36	3·54	0·58	0·07
Vitamin E (mg)	2·23	2·49	3·12	3·95	0·39
Dietary folate equivalents (Mcg)	99·12	32·53	101·87	63·03	0·86
Ca (mg)	531·66	128·34	603·34	102·36	0·05*
Fe (mg)	3·03	1·11	2·78	1·39	0·51
K (mg)	964·71	172·86	925·29	85·25	0·33

* Results were considered significant for *P* < 0·05.

**Table 6:** Comparison of macro- and micronutrients from mock 2016 to 2020 menu

Nutrient	Mean serving mock 2016 menu	sd	Mean serving 2020 menu	sd	*P*-value
Energy (kJ)	2170.24	259.7	2095.35	297.19	0·38
Protein (g)	26·55	3·08	24·71	3·29	0·06
Carbohydrate (g)	62·98	8·20	62·74	8·61	0·93
Dietary fibre (g)	5·70	1·72	5·67	1·69	0·96
Sugar (g)	35·41	7·48	35·64	9·05	0·93
Added sugar (g)	6·75	5·08	6·61	8·43	0·95
Total fat (g)	19·05	5·53	18·27	7·11	0·69
Saturated fat (g)	6·66	2·01	6·46	1·56	0·73
Monounsaturated fat (g)	5·82	2·15	4·79	1·42	0·07
Polyunsaturated fat (g)	4·96	3·61	5·62	4·17	0·58
Trans fat (g)	0·36	0·20	0·37	0·08	0·89
Cholesterol (mg)	51·87	21·14	41·34	8·81	0·03*
Na (mg)	825·53	218·78	627·88	164·65	0·002*
Vitamin A (mcg)	404·40	266·28	501·28	275·87	0·24
Vitamin C (mg)	23·43	31·46	23·87	26·73	0·96
Vitamin D (mcg)	3·25	0·36	3·54	0·58	0·06
Vitamin E (mg)	2·70	2·25	3·12	3·95	0·68
Dietary folate equivalents (Mcg)	80·28	35·04	101·87	63·03	0·18
Ca (mg)	541·25	128·87	603·34	102·36	0·09
Fe (mg)	2·65	0·82	2·78	1·39	0·73
K (mg)	1004·28	180·30	925·29	85·25	0·07

* Results were considered significant for *P* < 0·05.

Table 5 shows descriptive statistics and significant differences in macro- and micronutrients in grams and micrograms between the 2016 and 2020 menus. Among macronutrients, there were significant reductions in carbohydrates (*P* = 0·03) and cholesterol (*P* = 0·04). Among micronutrients, there was a significant reduction in Na (*P* = 0·002). There were significant increases in vitamin A (*P* = 0·05) and Ca (*P* = 0·05).

Among HEI-2015 adequacy components, there were reductions in the mean servings of total fruit, whole fruit, total protein foods and fatty acids between 2016 and 2020 (See Table 7). There were significant increases in whole grains (*P* = 0·003) and total vegetables (*P* < 0·001). Among moderation components, there were significant reductions in refined grains (*P* = 0·001) and Na (*P* = 0·02).

**Table 7:** Comparison of HEI food components in cup and ounce equivalents by density from 2016 to 2020 menu

Food component	Mean serving 2016 menu	sd	Mean serving 2020 menu	sd	*P*-value
	Adequacy components				
Total fruit (cup equiv per 1000 kcal)	0·98	0·44	0·89	0·27	0·42
Whole fruit (cup equiv per 1000 kcal)	0·66	0·43	0·54	0·48	0·41
Total vegetables (cup equiv per 1000 kcal)	0·60	0·34	1·01	0·28	< 0·001*
Dark green vegetables (cup equiv per 1000 kcal)	0·05	0·18	0·30	0·35	0·006*
Whole grains (oz equiv per 1000 kcal)	1·12	0·92	2·00	0·91	0·003*
Dairy (cup equiv per 1000 kcal)	2·58	0·98	3·11	0·93	0·08
Total protein foods (oz equiv per 1000 kcal)	3·63	1·87	2·61	1·76	0·07
Seafood and plant proteins (oz eq per 1000 kcal)	0·71	1·23	1·11	1·33	0·30
Fatty acids (fatty acid ratio)	1·77	0·84	1·58	0·60	0·39
	Moderation components				
Refined grains (oz equiv per 1000 kcal)	2·52	1·58	1·25	0·69	0·001*
Na (g per 1000 kcal)	1·56	0·45	1·27	0·34	0·02*
Added sugars (% kcal from added sugars)	5·84	4·01	5·27	6·78	0·74
Saturated fats (% SFA)	10·81	2·75	11·34	1·85	0·47

* Results were considered significant for *P* < 0·05.

In our robustness check, Table 6 shows that when accounting for menu changes that would have occurred irrespective of SRCP intervention, macronutrient trends in cholesterol reduction (*P* = 0·03) and micronutrient trends in Na reduction (*P* = 0·002) remained. Table 8 shows that HEI-2015 adequacy component trends in total vegetables (*P* = 0·008) and the addition of a significant increase in dark green vegetables (*P* = 0·007). There was also a significant reduction in total protein foods (*P* = 0·05). Among moderation components, the trend remained for a significant reduction in Na (*P* = 0·01).

**Table 8:** Comparison of Healthy Eating Index food components in cup and ounce equivalents per 1000 calories from mock baseline to year 5

Food Component	Mean serving mock 2016 menu	sd	Mean serving 2020 menu	sd	*P*-value
	Adequacy components				
Total fruit (cup equiv per 1000 kcal)	0·86	0·33	0·89	0·27	0·68
Whole fruit (cup equiv per 1000 kcal)	0·65	0·47	0·54	0·48	0·47
Total vegetables (cup equiv per 1000 kcal)	0·78	0·25	1·01	0·28	0·01*
Dark green vegetables (cup equiv per 1000 kcal)	0·05	0·20	0·30	0·35	0·007*
Whole grains (oz equiv per 1000 kcal)	2·21	1·16	2·00	0·91	0·52
Dairy (cup equiv per 1000 kcal)	2·70	1·07	3·11	0·93	0·19
Total protein foods (oz equiv per 1000 kcal)	3·77	1·95	2·61	1·76	0·05*
Seafood and plant proteins (oz eq per 1000 kcal)	0·70	1·21	1·11	1·33	0·29
Fatty acids (fatty acid ratio)	1·74	0·83	1·58	0·60	0·47
	Moderation components				
Refined grains (oz equiv per 1000 kcal)	1·17	1·01	1·25	0·69	0·77
Na (g per 1000 kcal)	1·60	0·46	1·27	0·34	0·01*
Added sugars (% kcal from added sugars)	5·42	4·54	5·27	6·78	0·93
Saturated fats (% SFA)	11·36	2·97	11·34	1·85	0·98

* Results were considered significant for *P* < 0·05.

## Discussion

Implementing Na reduction strategies on a 4-week ARASM menu cycle resulted in a significant reduction in Na from 2016 to 2020 with a nominal impact on other HEI-2015 adequacy or moderation food components. While there was an increase in whole grain servings and reduction in refined grains between 2016 and 2020, these significant differences did not remain in the robustness check that accounted for changes that would have been made based on the requirement for whole grain products in the revised CACFP meal pattern guidelines. There was a significant increase in total vegetables and dark green vegetables that remained when using either the true 2016 or mock 2016 dataset in the analysis. This indicates these menu changes occurred because of the SRCP intervention activities. Significant reductions in Na did not result in a significant increase in any HEI-2015 moderation components. This is important to note specifically for saturated fat and added sugars because they are used alongside Na by food manufacturers to maintain sensory appeal and increase shelf life in packaged foods^(25)^.

Na reduction interventions had a minimal impact on macro- and micronutrients beyond a significant reduction in Na and cholesterol. A significant increase in vitamin A was seen between the 2016 and 2020 results, but it did not remain after adjusting the servings of vegetables to meet CACFP meal pattern updates in the robustness check. These results indicate that it is possible to reduce Na in CACFP meals without increasing sugar and fat, or decreasing commonly under-consumed nutrients, such as fibre, potassium, vitamin A and vitamin D^(9)^. These findings occurred in a CACFP programme that relies on prepackaged foods due to cold storage and meal site needs, demonstrating the ability to improve the nutrition profile with significant programmatic restraints.

The mock 2016 dataset, which represents a pre-Na reduction intervention menu, was compliant with the revised CACFP meal pattern. This demonstrates that CACFP meal pattern adherence can still result in significant variation in nutrient composition, including high levels of Na. Prior studies have found that Na content often exceeds the daily limits across age groups in CACFP meals both after the revised CACFP meal pattern and after interventions^(16,18,19,26)^. These findings align with a health impact assessment, which found that the new standards would have an uncertain effect on Na content^(9)^. This study showed that the CACFP meal pattern rule change alone did not reduce Na, but that it is possible to focus interventions on reducing Na. Additional research is needed to assess the feasibility of placing limits on Na content either through meal pattern requirements or best practice recommendations.

Sugar has been limited in CACFP meals through meal pattern requirements and best practices and are an example of possible actions to limit Na. The sugar limits on dry cereal and yogurt and prohibition of reimbursement for grain-based desserts demonstrate that it may be feasible to implement a similar policy for frequently consumed high-Na foods such as lunch meat, cheese, bread and frozen entrees.

Obtaining staff buy-in from the food service company and Indy Parks staff prior to the intervention helped to facilitate success. The contract through which the food service company was compensated may have further increased buy-in and willingness to trial lower Na products. While the cost of two product swaps and three meal replacements were higher than the items they replaced, the food service company was able to balance this through cost savings on other products. The SRCP RDN provided technical assistance in identifying new products, organising taste tests and demonstrating child acceptance of new items, limiting the additional staff burden for Indy Parks and the food service company. The Indy Parks ARASM programme manager and the food service company administrator and chef attended quarterly meetings, but there was no additional labour for meal site staff or the food service company line staff. This programme’s requirement of using individually packaged meals impacted the types of product swaps and meal replacements that were possible as other strategies that have been shown to reduce Na, such as scratch cooking, were not able to be used^(18,27)^.

The findings of this study should be considered within its limitations. One limitation of this study is that the menu from one CACFP programme was analysed. Nutrition information came from nutrition facts information of foods on the menu as opposed to foods consumed by participants. An additional limitation is that because this CACFP programme serves only one meal per day, the authors could not calculate an HEI-2015 score. In this project, RDN were able to conduct annual nutrient analyses and provide consultation with food service providers, which facilitated Na reduction. Without adequate funding and RDN expertise, other CACFP providers may not have the capacity to replicate these results.

This is one of the few studies on an intervention that sought to reduce a specific nutrient in CACFP meals. Findings from this study can inform future interventions focussed on reducing Na in CACFP meals. Future studies should include an increased number and variety of CACFP sites, including childcare centers and family childcare homes.

### Conclusion

Meals served through CACFP often exceed the recommended limits for Na content, despite being compliant with the meal pattern. This study demonstrates that Na content can be reduced in CACFP meals without compromising the nutritional quality of meals served, but providers may need additional technical assistance and guidance to achieve this. Future studies are needed to identify feasible best practices and policies to reduce Na content in the CACFP meal pattern.
